# Toward Dependable Internet of Medical Things: IEEE 802.15.6 Ultra-Wideband Physical Layer Utilizing Superorthogonal Convolutional Code

**DOI:** 10.3390/s22062172

**Published:** 2022-03-10

**Authors:** Kento Takabayashi, Hirokazu Tanaka, Katsumi Sakakibara

**Affiliations:** 1 Department of Information and Communication Engineering, Faculty of Computer Science and Systems Engineering, Okayama Prefectural University, Okayama 719-1197, Japan; sakaki@c.oka-pu.ac.jp; 2 Graduate School of Information Sciences, Hiroshima City University, 3-4-1, Ozuka-Higashi, Asa-Minami-Ku, Hiroshima 731-3194, Japan; hi.tanaka@m.ieice.org

**Keywords:** wireless body area network, IEEE 802.15.6, UWB, superorthogonal convolutional code, error controlling scheme

## Abstract

Wireless body area networks (WBANs) are attracting attention as a very important technology for realizing an Internet of Medical Things (IoMT). IEEE 802.15.6 is well known as one of the international standards for WBANs for the IoMT. This article proposes the combination of the IEEE 802.15.6 ultra-wideband (UWB) physical layer (PHY) with a super orthogonal convolutional code (SOOC) and evaluates its performance as a dependable WBAN. Numerical results show that sufficient dependability cannot be obtained with the error-correcting code specified in IEEE 802.15.6 when applying the single pulse option, while both high energy efficiency and dependability can be obtained by applying an SOCC. In addition, it is confirmed that higher dependability can be obtained by combining an SOCC with a Reed–Solomon (RS) code with a coding rate that is almost the same as the error correction code specified in the standard. Furthermore, the results indicate that high dependability and energy efficiency can be obtained by adjusting the SOCC coding rate and UWB PHY parameters, even in the burst pulse option. The SOCC-applied UWB PHY of this research satisfies the high requirements of the IoMT.

## 1. Introduction

With the evolution of technologies such as sensing, edge computing, and artificial intelligence (AI), the Internet of Things (IoT) is recognized as a highly important societal technology. Applications using IoT technology are widely deployed. In particular, the Internet of Medical Things (IoMT) is attracting attention in terms of constructing home medical care and telemedicine systems using medical and healthcare devices and robots [1,2,3,4]. The system involves wearable, wireless vital sign sensors or medical robots. One type of technology supporting the development of the IoMT system is wireless body area networks (WBANs), which flexibly connect biosensors placed near the surface of the body [5,6,7,8,9,10,11,12,13,14]. A WBAN is a wireless network formed by connecting small sensors located on the surface, inside, and in the immediate vicinity of the body by wireless communication. Numerous studies regarding WBANs have been conducted. For example, an adaptive WBAN scheme reconfiguring a network by learning from body kinematics and biosignals was proposed in [13]. The authors of [14] proposed a novel energy-efficient medium access control (MAC) protocol for an in-body sensor-based WBAN that modified a superframe structure separating the access phases for an emergency event and a regular event. In [15], a marginal utility theoretical method was proposed to allocate radio resources to on/in-body sensors in a fair and efficient manner in a wirelessly powered body area network.

IEEE 802.15.6 was developed as an international standard primarily focused on implantable and wearable WBANs and is intended for use with the IoMT [16]. IEEE 802.15.6 includes a physical layer (PHY) that defines communication methods and radio wave specifications, a medium access control (MAC) layer that defines network setup and channel access methods, and security specifications [16]. This research mainly focuses on the PHY. There are multiple frequency bands that can be used by a WBAN, and IEEE 802.15.6 defines three PHYs to support these frequency bands: the narrowband (NB), the ultra-wideband (UWB), and human body communications (HBCs). In particular, this research focuses on an impulse radio UWB PHY (IR-UWB-PHY). Features of the UWB include high-speed communication utilizing the wide bandwidth, ultra-high-precision positioning capability, low energy consumption, high multipath resolution, and coexistence with existing wireless communication systems [17]. Hence, the UWB is a technology that may satisfy the requirements of the IoMT. However, the UWB PHY has a very low power spectral density emission limit ($G_{lim}$). For example, it is defined as $G_{lim}$ = −41.3 dBm/MHz according to regulations in the U.S. [18]. Hence, it is necessary to ensure the reliability of communications in the UWB PHY.

As a method of improving the reliability of wireless communication, the application of error correction codes can be considered. One example is super orthogonal convolutional codes (SOCCs) [19,20,21,22]. It is possible to design an error-correcting SOCC with a very low coding rate, resulting in this type of code having very strong error-correcting capabilities. SOCCs have attracted attention as error correction codes for code division multiple access (CDMA) using the spread spectrum [21,22]. Therefore, SOCCs are considered to be compatible with the UWB, as they use a very wide bandwidth. In related research, the authors of [23,24] evaluated the performance when SOCCs were applied in a UWB PHY. However, they did not consider the UWB PHY defined by IEEE 802.15.6 and did not target the WBAN channel model. Then, our previous research provided a quality of service (QoS) control scheme using decomposable error control codes for an IEEE 802.15.6-based WBAN [25]. However, the previous scheme is different from the scheme targeted in this research because retransmission is performed multiple times. In addition, the QoS control scheme is not specific to the IEEE 802.15.6 UWB PHY. Our previous research also evaluated a UWB PHY-based WBAN using a SOCC [26]. However, this research only assumed a payload and a simple additive white Gaussian noise (AWGN) channel model. Hence, a preamble, a header and a WBAN channel model were not considered. Reference [27] also studied SOCCs. However, this research targeted orthogonal frequency-division multiplexing (OFDM) and general machine-to-machine (M2M) communications, which is different from this article.

This research provides a novel performance evaluation of the transmission failure ratio and energy efficiency in the UWB PHY of IEEE 802.15.6 with the application of an SOCC through computer simulations. Numerical results show that this combination is very effective in the WBAN channel model referenced in [28]. In addition, the SOCC and UWB PHY parameters that satisfy the requirements are determined. Furthermore, the research supports the idea that the SOCC-applied UWB PHY can satisfy the high requirements of the IoMT and that it is possible to build a wireless network to realize the IoMT.

The remainder of this paper is organized as follows. In Section 2, the UWB PHY of IEEE 802.15.6 is summarized. In Section 3, an SOCC is introduced, and how to apply an SOCC in the IEEE 802.15.6 UWB PHY is explained. The numerical results of the performance evaluation are provided in Section 4. Conclusions and suggestions for future research are presented in Section 5.

## 2. Materials and Methods

### 2.1. IEEE 802.15.6 UWB PHY

#### 2.1.1. Operating Frequency Bands

**Figure 1:**
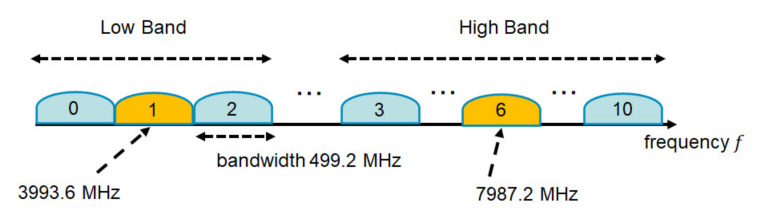
Operating frequency bands for IEEE 802.15.6 UWB PHY.

The UWB band is divided into two band groups: a low band (channels 0–2) and a high band (channels 3–10). The low band and high band are divided into operating frequency channels with 499.2 MHz bandwidth, as shown in Figure 1. A UWB device that implements the low band needs to support channel 1, whose center frequency is 3993.6 MHz. The remaining low-band channels are optional. On the other hand, a UWB device that implements the high band needs to support channel 6, whose center frequency is 7987.2 MHz. The remaining high-band channels are optional.

#### 2.1.2. UWB PHY Frame Format

**Figure 2:**
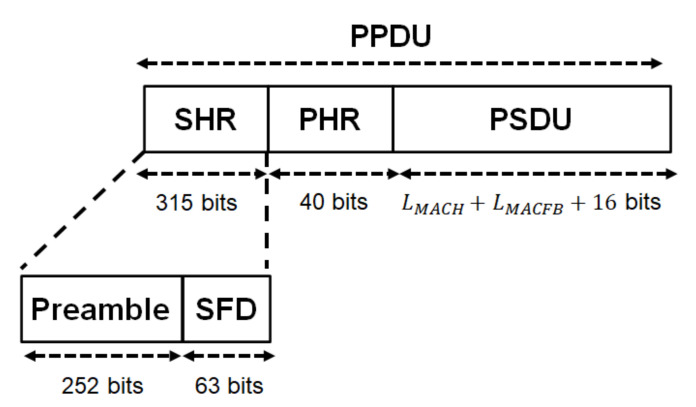
UWB PHY frame format.

The UWB PHY frame format is formed by the synchronization header (SHR), the physical layer header (PHR), and the physical layer service data unit (PSDU), as shown in Figure 2.

The SHR is divided into two parts. The first part is the preamble, intended for timing synchronization, packet detection, and carrier frequency offset recovery. Kasami sequences $C_{i}$ with a length of 63 are used to build the preamble referenced in [16]. The preamble consists of 4 repetitions of the symbol $S_{i}$. $S_{i}$ is computed as follows:

(1) $$S_{i}=C_{i}⊗\delta_{L}.$$

Here, $\delta_{L}$ is ${\left(1,0,0,\cdots ,0\right)}_{1\times L}$, and the operator ⊗ is the Kronecker product. L depends on the modulation. The second part is the start-of-frame delimiter (SFD) for frame synchronization. The SFD is formed based on the symbol $\bar{S_{i}}$. $\bar{S_{i}}$ represents an inversion of the i-th Kasami sequence bits $C_{i}$ in $S_{i}$. The SFD is chosen to have low cross-correlation with the preamble such that the transition of the correlation from preamble to SFD does not degrade the detection of the SFD. Therefore, the length of the SHR is 315 bits.

The PHR contains information about the data rate of the PSDU, length of the medium access control (MAC) frame body, pulse shape, burst mode, and so on. The PHR is encoded with cyclic redundancy check (CRC)-4 ITU as an error detection code. In addition, it is encoded by the (40, 28) shortened Bose–Chaudhuri–Hocquenghem (BCH) code derived from the (63, 51) BCH code in the default mode and the (91, 28) shortened BCH code derived from the (127, 64) BCH code in the high-quality of service (QoS) mode as an error-correcting code. This research assumes the default mode. Hence, the length of the PHR is 40 bits.

The PSDU contains the MAC protocol data unit (MPDU) and BCH parity bits in the default mode. The MPDU is defined as the concatenation of the MAC header, MAC frame body, and frame check sequence (FCS) whose lengths are $L_{MACH}$, $L_{MACFB}$, and 16 bits, respectively. Here, the MPDU is encoded by CRC-16-CCITT as an error detection code. In addition, the PSDU is encoded by the (63, 51) BCH code in the default mode.

#### 2.1.3. Modulation and Pulse Shaping

In the IR-UWB PHY, the bits of the physical layer protocol data unit (PPDU) are modulated by either on–off modulation or differentially encoded binary phase shift keying (DBPSK)/quadrature phase shift keying (DQPSK). This research assumes DBPSK because the modulation has higher robustness against errors than on–off modulation and can be more easily simulated [29,30,31]. The DBPSK transmitting symbols are given by the following equation:

(2) $$c_{m}=c_{m-1}\exp\left(j\varphi_{m}\right).$$

Here, $c_{m}$ is the m-th encoded DBPSK symbol, $m=\left(0,1,\ldots ,N\right)$, N gives the number of symbols, $c_{-1}=1$, and $\varphi_{0}$ is an arbitrary phase. The symbol $c_{0}$ serves as a phase reference for the differential encoding of the first bit. In the case of DBSPK modulation, the number of symbols is N=P, where P is the number of bits in the PPDU $\left(g_{0},g_{1},\cdots ,g_{P-1}\right)$. The symbol $c_{m}$ carries one bit of information. The mapping of information bits onto $\varphi_{m}$ is given in Table 1.

After the generation of the DBPSK symbols, pulse shaping places a pulse waveform according to the UWB symbol structure. The transmitting signal is given as follows:

(3) $$x\left(t\right)=\sum_{m=0}^{N}c_{m}w\left(t-mT_{sys}-h^{\left(m\right)}T_{w}\right).$$

Here, $T_{sys}$ is the symbol time, $h^{\left(m\right)}$ is the time-hopping sequence, $T_{w}$ is the pulse waveform duration, and $w\left(t\right)$ is the pulse waveform. $w\left(t\right)$ is expressed according to the pulse option as follows:

(4) $$w\left(t\right)=\left\{\begin{array}{c} p\left(t\right)\;\left(\mathrm{single}\;\mathrm{pulse}\;\mathrm{option},\;T_{w}=T_{p}\right)\\ \overset{N_{cpb}-1}{\underset{i=0}{\sum }}\left(1-2s_{i}\right)p\left(t-iT_{p}\right)\;\left(\mathrm{burst}\;\mathrm{pulse}\;\mathrm{option},\;T_{w}=N_{cpb}T_{p}\right) \end{array}\right.$$

**Figure 3:**
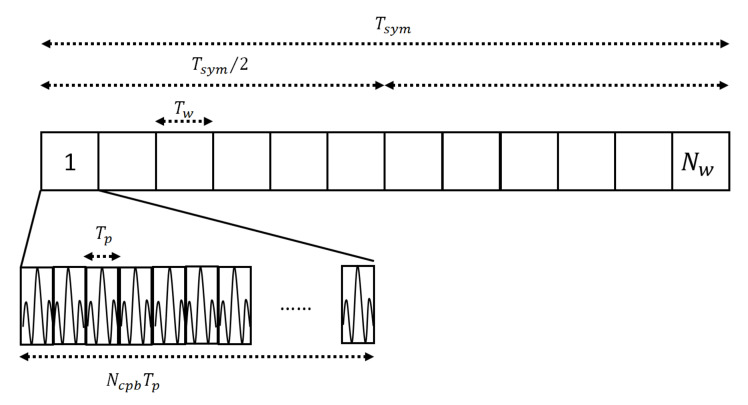
Signal transmission example where the burst pulse option is used.

Here, $s_{i}$ is given by the static scrambling sequences, $p\left(t\right)$ is a fixed pulse waveform, and $T_{p}$ is the duration of $p\left(t\right)$. The single pulse option is defined as a single pulse transmitted per symbol, while the burst pulse option is defined as a concatenation of pulses transmitted per symbol. The burst pulse option can be used to reduce the data rate while improving the received power by correlating multiple pulses. Figure 3 illustrates an example of signal transmission when the burst pulse option is used.

### 2.2. SOCC

#### 2.2.1. SOCC Encoder Configuration

**Figure 4:**
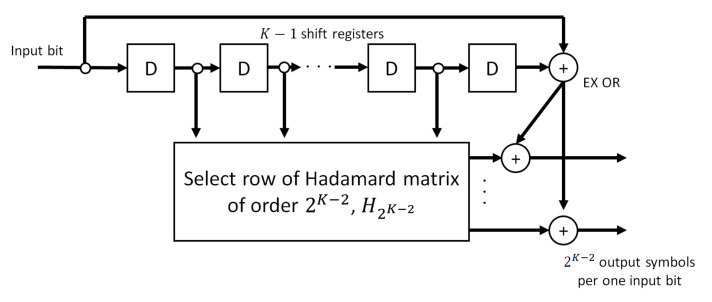
SOCC encoder.

An SOCC is a kind of orthogonal convolutional code with a very low coding rate [19,20,21,22]. The encoder of an SOCC is illustrated in Figure 4. The SOCC encoder consists of K−1 shift registers and a block orthogonal encoder with a Hadamard matrix of order $2^{K-2}$. Here, K represents the constraint length. The encoder maps the bits stored in the shift registers up to K−2th to the Hadamard sequence with a sequence length of $2^{K-2}$. Then, the SOCC symbols are generated by applying an exclusive-OR to the output of the Hadamard sequences, the input bit and the K−1th stored bit. The coding rate $R_{SOCC}$ of an SOCC is $2^{-\left(K-2\right)}$. Viterbi decoding can be used to decode SOCCs as well as general convolutional codes.

#### 2.2.2. Application of SOCCs to IEEE 802.15.6 UWB PHY

**Figure 5:**
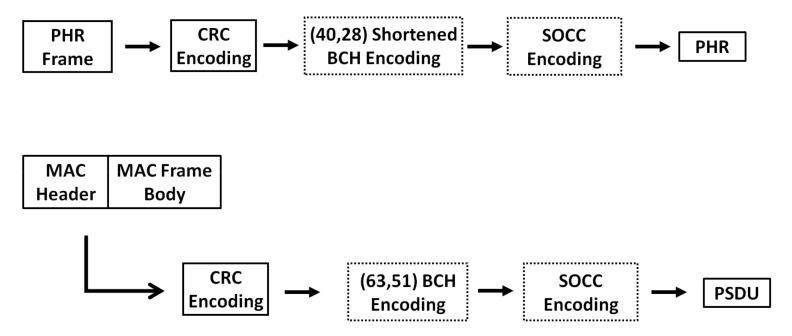
Block diagrams showing the procedure for applying an SOCC to the IEEE 802.15.6 UWB PHY.

Figure 5 shows a block diagram of the procedure for applying SOCCs to the IEEE 802.15.6 UWB PHY. For the PHR, after the CRC encoding of the PHR frame, whether to apply the shortened BCH code is determined, and then, whether to apply an SOCC is determined. Similarly, for the PSDU, after the CRC encoding of the MAC header and MAC frame body, whether to apply the BCH code is determined, and then, whether to apply an SOCC is determined. In other words, concatenated encoding using the BCH code as the outer code and an SOCC as the inner code is performed when both the BCH code and SOCC are applied.

## 3. Results

### 3.1. Computer Simulation Parameters

This section describes the performance of the transmission failure ratio and energy efficiency of the IR-UWB determined with computer simulations. The main parameters of the computer simulations are listed in Table 2. In addition to the AWGN channel model, the IEEE model CM 3, which targets wearable WBANs and includes multipath fading, is applied as a channel model [28]. For comparison, the computer simulation also evaluates the case where the (11, 5) shortened Reed–Solomon (RS) code and the (31, 25) RS code are applied to the PHR and PSDU, respectively, as error correction codes with coding rates almost equal to those of the BCH codes used in IEEE 802.15.6. The computer simulator was constructed in MATLAB.

The energy efficiency in the PHY is derived from [17,32,33,34,35] as follows:

(5) $$\eta \equiv \frac{P_{succ}L_{info}}{E_{link}}$$

In Equation (5), $L_{info}$ is the information bit length in the PSDU, $E_{link}$ is the energy consumption of the communication link, and $P_{succ}$ is the transmission success ratio. $P_{succ}$ can be expressed as follows [32]:

(6) $$P_{succ}=1-P_{fail}=\left(1-P_{fail,\;preamble}\right)\left(1-P_{e,\;PLCPheader}\right)\left(1-P_{e,\;PSDU}\right)$$

In Equation (6), $P_{fail}$ is the transmission failure ratio, $P_{fail,\;preamble}$ is the SFD detection failure ratio, $P_{e,\;PHR}$ is the PHR error ratio, and $P_{e,\;PSDU}$ is the PSDU error ratio. Additionally, $E_{link}$ can be simply described as follows [32]:

(7) $$E_{link}=L_{PPDU}\left(P_{tx}+P_{rx}\right)/T_{w}+\left(\varepsilon_{enc}+\varepsilon_{dec}\right)$$

(8) $$L_{PPDU}=L_{SHR}+L_{PHR}+L_{PSDU}$$

In Equations (7) and (8), $L_{PPDU}$, $L_{SHR}$, $L_{PHR}$, and $L_{PSDU}$ are the lengths of the PPDU, the SHR, the PHR, and the PSDU, respectively; $P_{tx}$ and $P_{rx}$ are the transmitter and receiver power consumptions, respectively; and $\varepsilon_{enc}$ and $\varepsilon_{dec}$ are the encoding and decoding energies, respectively [17,32,34].

### 3.2. Single Pulse Option

**Figure 6:**
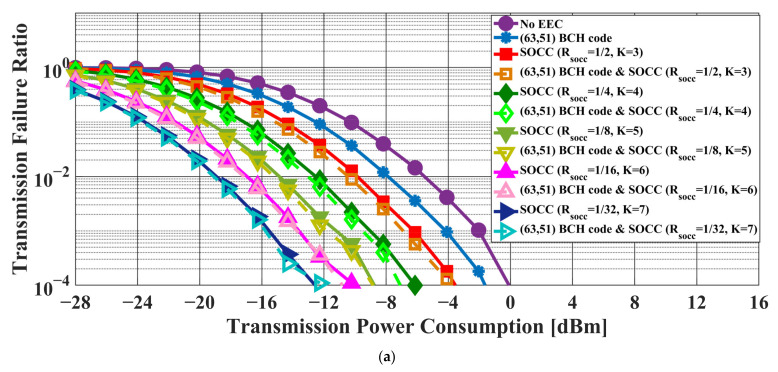

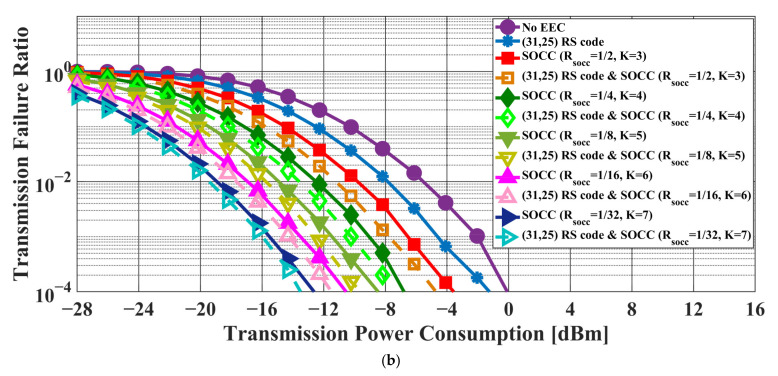
Transmission failure ratio as a function of the transmission power in the case of the AWGN channel model. (**a**) BCH coding case. (**b**) RS coding case.

**Figure 7:**
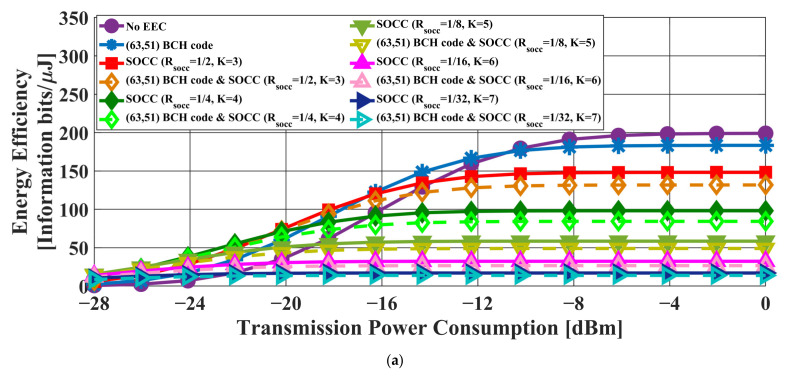

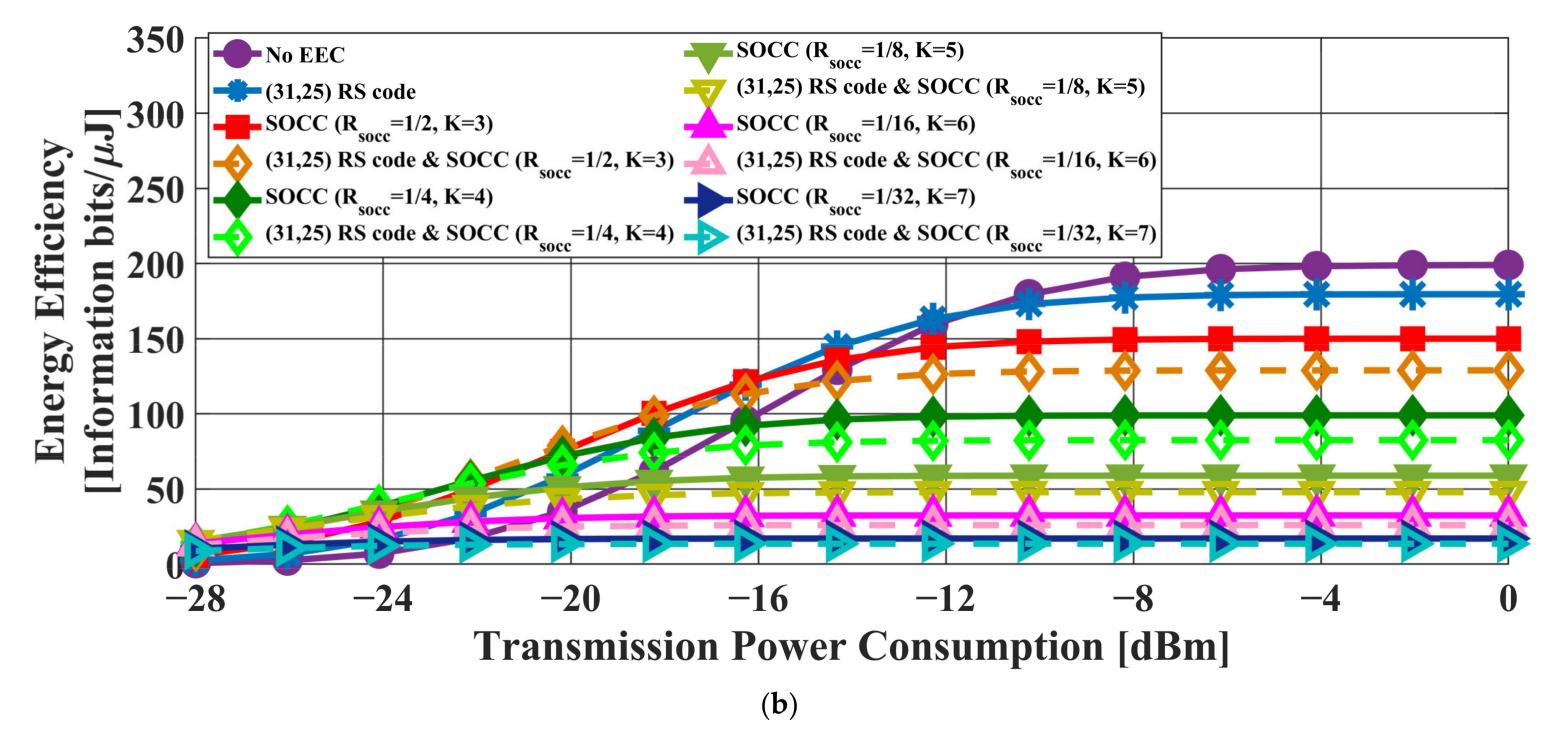
Energy efficiency as a function of the transmission power in the case of the AWGN channel model. (**a**) BCH coding case. (**b**) RS coding case.

**Figure 8:**
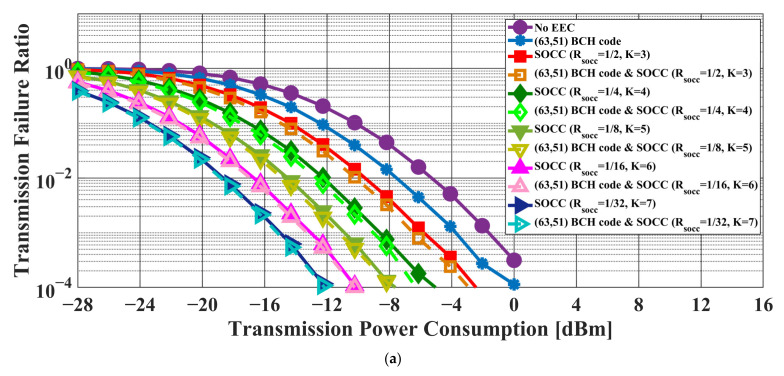

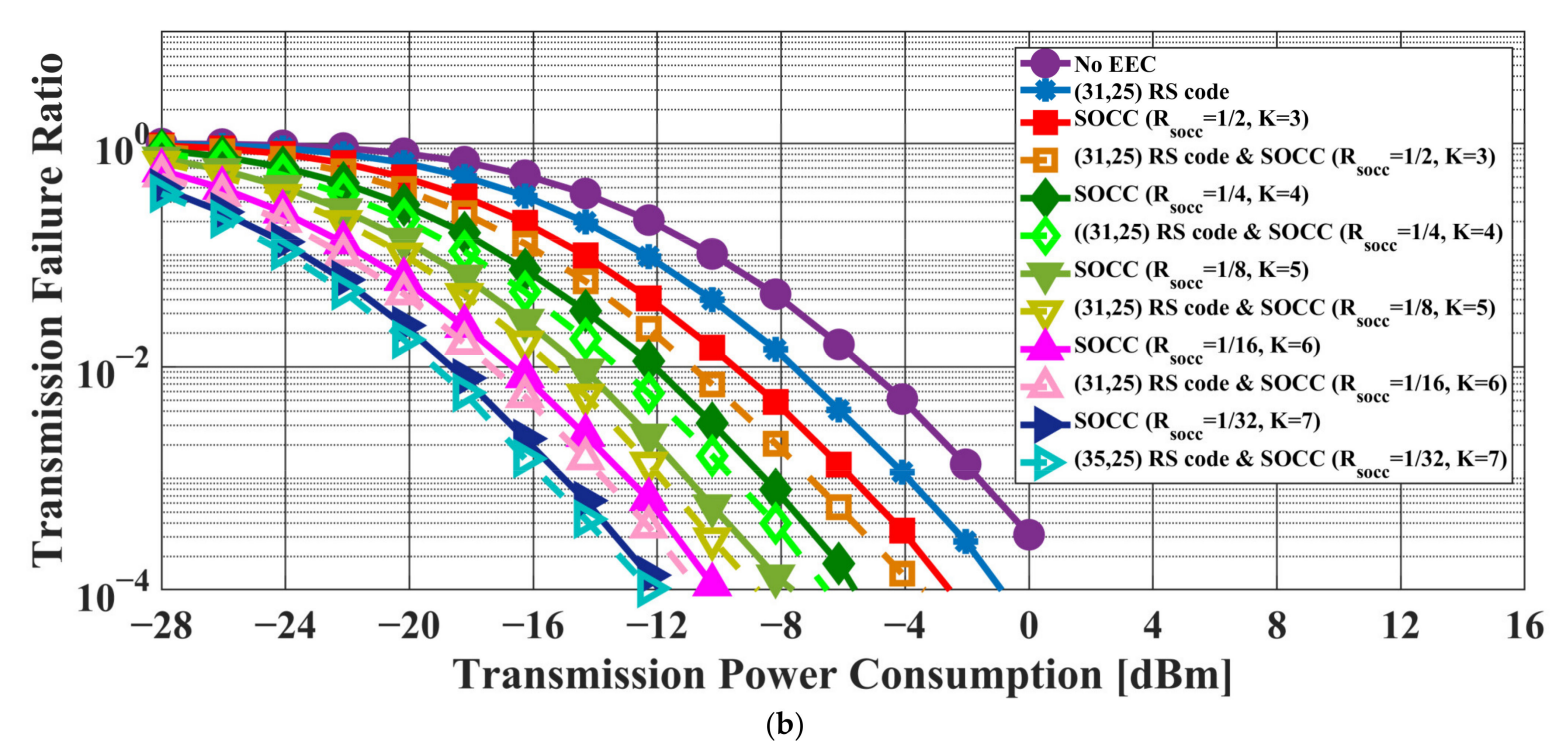
Transmission failure ratio as a function of the transmission power in the case of the IEEE model CM3. (**a**) BCH coding case. (**b**) RS coding case.

**Figure 9:**
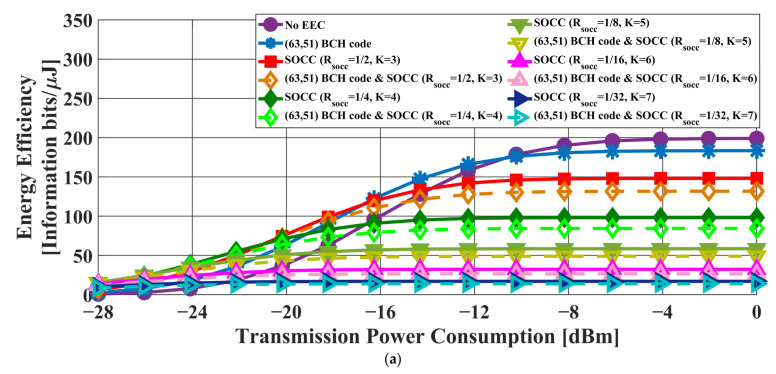

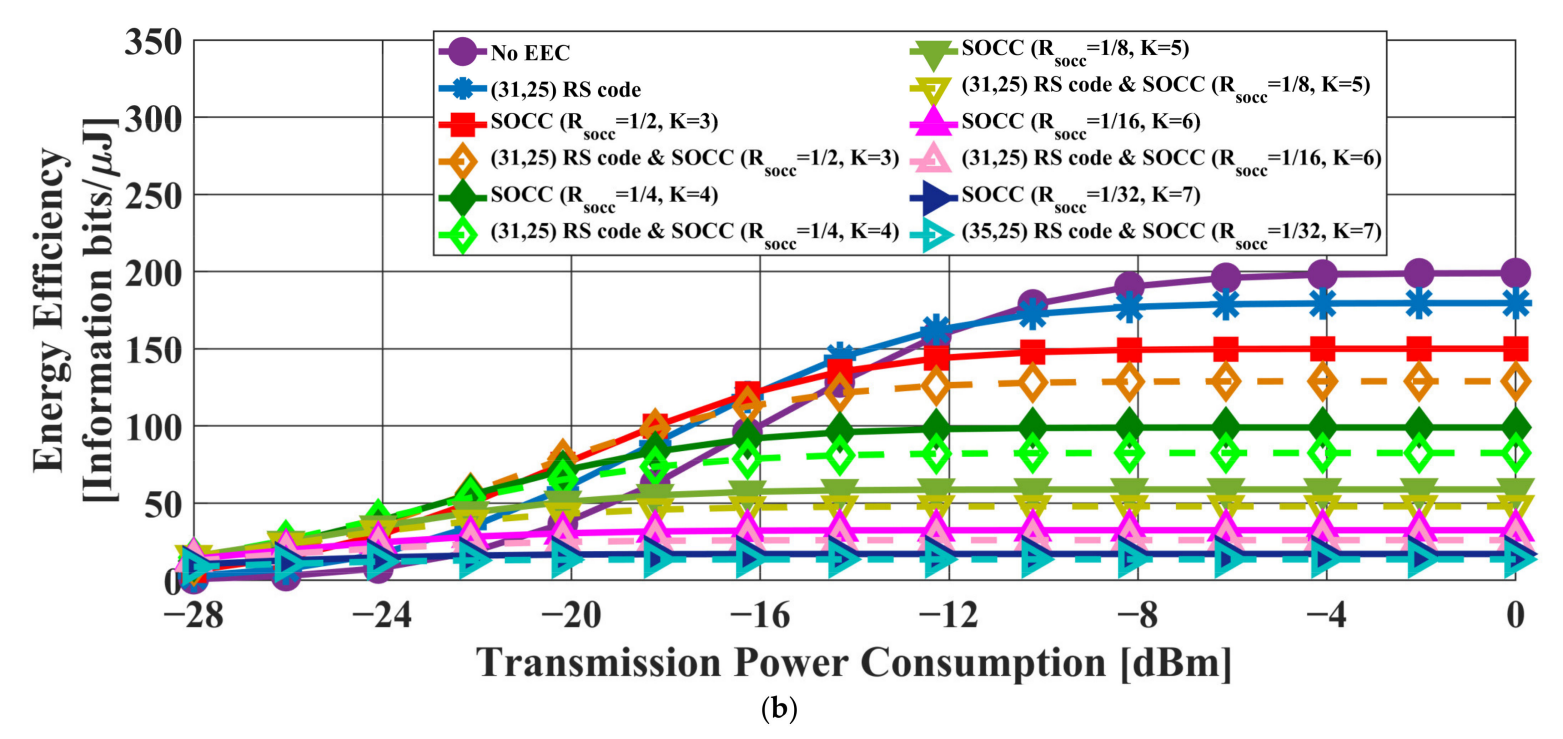
Energy efficiency as a function of the transmission power in the case of the IEEE model CM3. (**a**) BCH coding case. (**b**) RS coding case.

Figure 6 and Figure 7 present the transmission failure ratio and energy efficiency, respectively, as a function of transmission power in the case of the AWGN channel model. In addition, Figure 8 and Figure 9 show the transmission failure ratio and energy efficiency, respectively, as a function of the transmission power in the case of the IEEE model CM3.

The overall tendency was for the performance of the IEEE model CM3 to deteriorate compared to that of the AWGN channel due to the effect of multipath fading. When the constraint length of the SOCC increased, the transmission failure ratio improved because the error-correcting capability increased with decreasing $R_{SOCC}$. On the other hand, the energy efficiency decreased because the number of redundant bits in the PPDU increased.

There was almost no difference between the applications of the concatenated coding of BCH codes and an SOCC and a single SOCC when $R_{SOCC}$ = 1/8 or less in the former. On the other hand, an improvement effect was seen even at $R_{SOCC}$ = 1/32 in the case of the concatenated coding of RS codes and an SOCC.

From the above, it was confirmed that the reliability of the IEEE 802.15.6 error control method was insufficient and that the application of an SOCC was effective when the single pulse option was applied.

### 3.3. Burst Pulse Option

**Figure 10:**
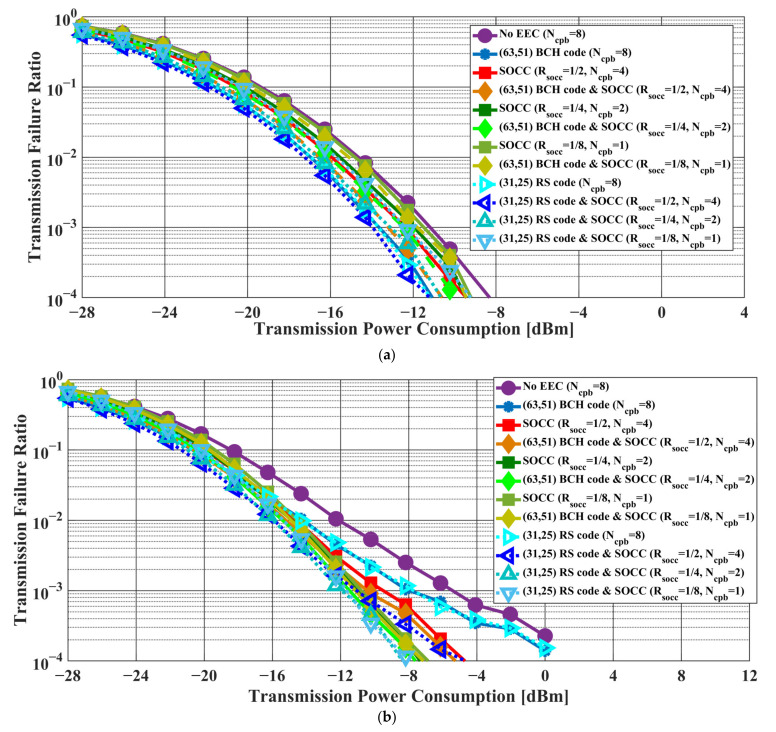
Transmission failure ratio as a function of the transmission power ($G=N_{cpb}R_{SOCC}^{-1}=8$). (**a**) AWGN channel model. (**b**) IEEE model CM3.

**Figure 11:**
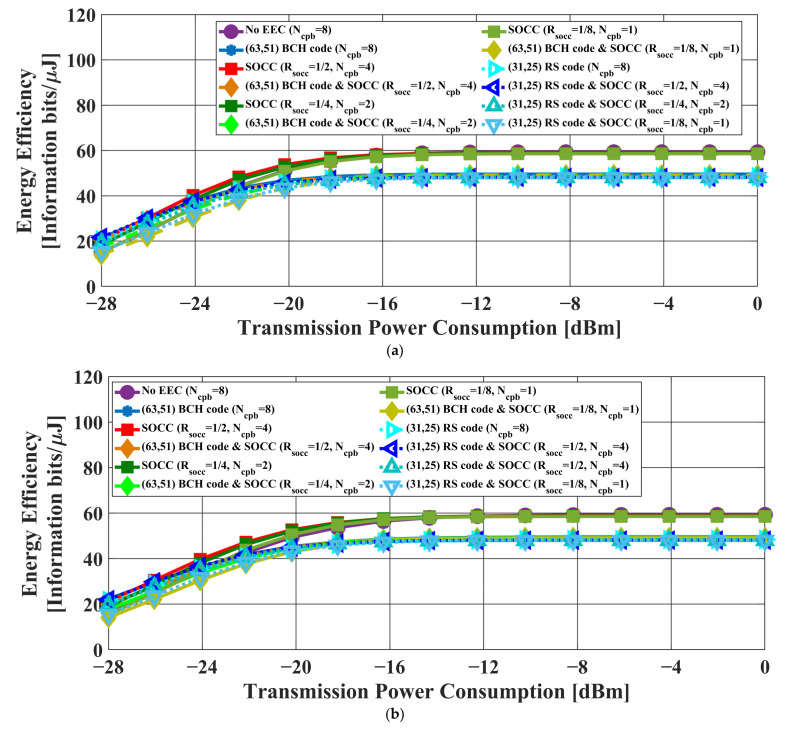
Energy efficiency as a function of the transmission power ($G=N_{cpb}R_{SOCC}^{-1}=8$). (**a**) AWGN channel model. (**b**) IEEE model CM3.

Figure 10 and Figure 11 present the transmission failure ratio and energy efficiency, respectively, as a function of the transmission power in the case of the AWGN channel model and IEEE model CM3. Here, the processing gain $G=N_{cpb}R_{SOCC}^{-1}$ was fixed at eight.

First, the transmission failure ratio was better when only an SOCC was applied than in the case without error-correcting codes in both channel models.

Next, the AWGN channel model and the IEEE model CM3 tended to differ in terms of transmission failure ratio performance. In the AWGN channel model, the effect of improving the transmission failure rating by BCH or RS coding was large. In particular, the best performance was obtained when an SOCC was applied with $R_{SOCC}$ = 1/2 and RS-encoded. In other words, simply lowering the SOCC coding rate did not provide good performance because erroneous corrections increase in frequency when decoding an SOCC in case sufficient received power cannot be obtained.

In the IEEE model CM3, the effect of improving the transmission failure ratio with an SOCC was substantial. In particular, better performance was obtained when an SOCC was applied with $R_{SOCC}\leq$ 1/4 and RS-encoded.

The energy efficiency was the highest when only an SOCC was applied and $R_{SOCC}$ = 1/2. In particular, the performance under these conditions was better than that when an error-correcting code was not utilized. This means that the improvement in the error-correcting capabilities had a greater effect than the energy consumption of SOCC encoding and decoding. On the other hand, the transmission failure ratio improved while the energy efficiency decreased when a BCH or an RS code and an SOCC were concatenated-encoded due to the increase in the number of redundant bits.

From the above, it was also confirmed that the application of an SOCC was effective when the burst pulse option was applied under fixed processing gain.

## 4. Discussion

This section discusses the evaluation results. In particular, the transmission failure ratio is focused on when meeting the UWB usage limitation such that BW = 499.2 MHz and $G_{lim}$ = −41.3 dBm/MHz (transmission power of −14.3 dBm).

First, Table 3, Table 4, Table 5 and Table 6 present the transmission failure ratio using an AWGN channel model and an IEEE model CM3 in the case of BCH and RS encoding when applying a single pulse option. Under the above conditions, the transmission failure ratio was more than $10^{-1}$ unless an SOCC was applied. This means that sufficient reliability cannot be ensured by the current standard in the case of the single pulse option. Then, the transmission failure ratio was below $10^{-1}$ when $R_{SOCC}$ = 1/2 and below $10^{-2}$ when $R_{SOCC}$ = 1/8, as shown in the tables. Furthermore, the energy efficiency when $R_{SOCC}$ = 1/2 was higher than that without error-correcting coding under the same conditions. In other words, reliability can be ensured while maintaining high energy efficiency by applying an SOCC.

Next, the comparison between BCH and RS encoding is discussed. Basically, RS encoding obtained better results. One of the reasons for this is that the compared RS code has better error-correcting capabilities than the BCH code specified in the standard. Another reason is that the RS code can be expected to correct burst errors that occur during SOCC decoding since the RS code is a burst error-correcting code. For the above reasons, effective improvement in the transmission failure ratio is achieved.

Table 7 and Table 8 show the transmission failure ratio using an AWGN channel model and an IEEE model CM3 in the case of BCH and RS encoding when applying a burst pulse option. As shown in the tables, the transmission failure ratio was less than $10^{-2}$ when the regulation for UWB use was satisfied except in the cases without an error-correcting code and with only BCH encoding in the IEEE model CM3. Using an AWGN channel, the case applying an SOCC ($R_{SOCC}$ = 1/2) and RS encoding has the best performance. This is due to the sufficient received power, the random error correction capability of the SOCC, and the addition of RS encoding with error correction capability. On the other hand, the case applying an SOCC ($R_{SOCC}$ = 1/4) and RS encoding has the best performance using the IEEE model CM3. The reason for this is that $T_{w}$ increases as $N_{cpb}$ increases according to Equation (4) in the case of the burst pulse option. Hence, the effect of multipath delay cannot be ignored, leading to performance degradation.

The above discussions clarify the effectiveness of applying an SOCC to WBANs when complying with the current regulations on the UWB. In addition, the optimal combination of IEEE 802.15.6 UWB PHY parameters $N_{cpb}$ and SOCC coding rate $R_{SOCC}$ were also clarified. These findings have not been shown in the existing studies mentioned in the introduction and are considered to contribute to the realization of a dependable IoMT utilizing UWB-based WBANs.

## 5. Conclusions

This research proposed applying an SOCC to the UWB PHY of IEEE 802.15.6 to improve its dependability and evaluated the effect of applying an SOCC by computer simulations. Numerical results showed that sufficient dependability could not be ensured by the error control method defined by IEEE 802.15.6 and that high energy efficiency was obtained while ensuring a sufficient transmission failure ratio by applying an SOCC in the single pulse option. In addition, it was confirmed that a combination effect could be obtained with an SOCC by applying RS codes with almost the same coding rate as the BCH code specified in IEEE 802.15.6. It was shown that high dependability and high energy efficiency could be obtained by applying an SOCC while utilizing the high multipath resolution of the UWB in the burst pulse option.

For future work, performance of SOCC applications in a wearable WBAN in a dynamic channel model environment will be evaluated. In addition, the optimization of the access protocol should be considered in this case.

## Figures and Tables

**Table 1:** Mapping of information bits onto $\varphi_{m}$ for DBPSK.

$g_{m}$	$\varphi_{m}$
0	0
1	π

**Table 2:** Computer simulation parameters.

Parameter	Details
Channel model	AWGN, IEEE model CM3
Path loss model	IEEE model CM3
Center frequency	3993.6 MHz
Bandwidth (BW)	499.2 MHz
Modulation	DBPSK
FEC (PHR)	(40, 28) shortened BCH code, (11, 5) shortened Reed–Solomon (RS) code, SOCC (K=3~7)
FEC (PSDU)	(63, 51) BCH code, (31, 25) Reed–Solomon (RS) code, SOCC (K=3~7)
Maximum transmission power	0 dBm
Thermal noise density $\left(N_{0}\right)$	−174 dBm/Hz
Implementation loss $\left(I_{\mathrm{dB}}\right)$	3 dB
Receiver noise figure $\left(NF_{\mathrm{dB}}\right)$	5 dB
Information bit length $\left(L_{info}\right)$	540 bits
Communication distance (d)	0.5 m
Duration of $p\left(t\right)$ $\left(T_{p}\right)$	2.003 ns
Pulse option	Single pulse option, burst pulse option
Uncoded symbol rate of the single pulse option $\left(R_{S}\right)$	7.8 Msps
$N_{cpb}$ (burst pulse option)	1, 2, 4, 8

**Table 3:** Transmission failure ratio using an AWGN channel model in the case of a single pulse option and BCH encoding. The transmission power is −14.3 dBm.

Channel Encoding	Transmission Failure Ratio
No BCH encoding	0.3501
BCH encoding	0.1891
SOCC $R_{SOCC}$ = 1/2	0.09212
SOCC $R_{SOCC}$ = 1/2 and BCH encoding	0.07295
SOCC $R_{SOCC}$ = 1/4	0.02656
SOCC $R_{SOCC}$ = 1/4 and BCH encoding	0.02078
SOCC $R_{SOCC}$ = 1/8	0.00709
SOCC $R_{SOCC}$ = 1/8 and BCH encoding	0.00558
SOCC $R_{SOCC}$ = 1/16	0.00177
SOCC $R_{SOCC}$ = 1/16 and BCH encoding	0.00149
SOCC $R_{SOCC}$ = 1/32	0.00037
SOCC $R_{SOCC}$ = 1/32 and BCH encoding	0.00025

**Table 4:** Transmission failure ratio using an AWGN channel model in the case of a single pulse option and RS encoding. The transmission power is −14.3 dBm.

Channel Encoding	Transmission Failure Ratio
No RS encoding	0.3501
RS encoding	0.1929
SOCC $R_{SOCC}$ = 1/2	0.09212
SOCC $R_{SOCC}$ = 1/2 and RS encoding	0.05422
SOCC $R_{SOCC}$ = 1/4	0.02656
SOCC $R_{SOCC}$ = 1/4 and RS encoding	0.01566
SOCC $R_{SOCC}$ = 1/8	0.00709
SOCC $R_{SOCC}$ = 1/8 and RS encoding	0.00403
SOCC $R_{SOCC}$ = 1/16	0.00177
SOCC $R_{SOCC}$ = 1/16 and RS encoding	0.00104
SOCC $R_{SOCC}$ = 1/32	0.00037
SOCC $R_{SOCC}$ = 1/32 and RS encoding	0.00025

**Table 5:** Transmission failure ratio using an IEEE model CM3 in the case of a single pulse option and BCH encoding. The transmission power is −14.3 dBm.

Channel Encoding	Transmission Failure Ratio
No BCH encoding	0.356
BCH encoding	0.1966
SOCC $R_{SOCC}$ = 1/2	0.09942
SOCC $R_{SOCC}$ = 1/2 and BCH encoding	0.07875
SOCC $R_{SOCC}$ = 1/4	0.03136
SOCC $R_{SOCC}$ = 1/4 and BCH encoding	0.02452
SOCC $R_{SOCC}$ = 1/8	0.00884
SOCC $R_{SOCC}$ = 1/8 and BCH encoding	0.00723
SOCC $R_{SOCC}$ = 1/16	0.00223
SOCC $R_{SOCC}$ = 1/16 and BCH encoding	0.00196
SOCC $R_{SOCC}$ = 1/32	0.00063
SOCC $R_{SOCC}$ = 1/32 and BCH encoding	0.00054

**Table 6:** Transmission failure ratio using an IEEE model CM3 in the case of a single pulse option and RS encoding. The transmission power is −14.3 dBm.

Channel Encoding	Transmission Failure Ratio
No RS encoding	0.356
RS encoding	0.1972
SOCC $R_{SOCC}$ = 1/2	0.09942
SOCC $R_{SOCC}$ = 1/2 and RS encoding	0.0584
SOCC $R_{SOCC}$ = 1/4	0.03136
SOCC $R_{SOCC}$ = 1/4 and RS encoding	0.01778
SOCC $R_{SOCC}$ = 1/8	0.00884
SOCC $R_{SOCC}$ = 1/8 and RS encoding	0.00555
SOCC $R_{SOCC}$ = 1/16	0.00223
SOCC $R_{SOCC}$ = 1/16 and RS encoding	0.00150
SOCC $R_{SOCC}$ = 1/32	0.00063
SOCC $R_{SOCC}$ = 1/32 and RS encoding	0.00043

**Table 7:** Transmission failure ratio using an AWGN channel model in the case of a burst pulse option and BCH and RS encoding. $G=N_{cpb}R_{SOCC}^{-1}=8$. The transmission power is −14.3 dBm.

Channel Encoding	Transmission Failure Ratio
No encoding	0.00829
BCH encoding	0.00163
RS encoding	0.00175
SOCC $R_{SOCC}$ = 1/2	0.00357
SOCC $R_{SOCC}$ = 1/2 and BCH encoding	0.00238
SOCC $R_{SOCC}$ = 1/2 and RS encoding	0.00139
SOCC $R_{SOCC}$ = 1/4	0.00463
SOCC $R_{SOCC}$ = 1/4 and BCH encoding	0.00333
SOCC $R_{SOCC}$ = 1/4 and RS encoding	0.00216
SOCC $R_{SOCC}$ = 1/8	0.00759
SOCC $R_{SOCC}$ = 1/8 and BCH encoding	0.00641
SOCC $R_{SOCC}$ = 1/8 and RS encoding	0.00421

**Table 8:** Transmission failure ratio using an IEEE model CM3 in the case of a burst pulse option and BCH and RS encoding. $G=N_{cpb}R_{SOCC}^{-1}=8$. The transmission power is −14.3 dBm.

Channel Encoding	Transmission Failure Ratio
No encoding	0.0239
BCH encoding	0.0105
RS encoding	0.00984
SOCC $R_{SOCC}$ = 1/2	0.00872
SOCC $R_{SOCC}$ = 1/2 and BCH encoding	0.0064
SOCC $R_{SOCC}$ = 1/2 and RS encoding	0.00429
SOCC $R_{SOCC}$ = 1/4	0.00762
SOCC $R_{SOCC}$ = 1/4 and BCH encoding	0.00577
SOCC $R_{SOCC}$ = 1/4 and RS encoding	0.00412
SOCC $R_{SOCC}$ = 1/8	0.0089
SOCC $R_{SOCC}$ = 1/8 and BCH encoding	0.00721
SOCC $R_{SOCC}$ = 1/8 and RS encoding	0.00543

## Data Availability

Not applicable.
